# CO_2_ lattice laser reverses skin aging caused by UVB

**DOI:** 10.18632/aging.103063

**Published:** 2020-04-20

**Authors:** Hongyi Wang, Bingyu Guo, Qiang Hui, Feng Lin, Kai Tao

**Affiliations:** 1 Reconstructive and Plastic Surgery, General Hospital of Northern Theater Command, Shenyang, P.R.China

**Keywords:** skin, CO lattice laser ^2^, photoaging, *in vitro*, *in vivo*

## Abstract

The carbon dioxide (CO_2_) lattice laser has been successfully used to treat facial skin photoaging induced by UV light. In this study, we analyzed the effect of CO_2_ lattice laser irradiation on skin photoaging, and investigated the underlying mechanisms. Our results demonstrate that the laser promoted collagen synthesis and proliferation of primary human skin fibroblasts, inhibited cell senescence, and induced expression of superoxide dismutase (SOD) and the signaling protein SMAD3. In addition, this laser reversed cell cycle arrest and fibroblast apoptosis induced by UVB irradiation, and restored fibroblast proliferation inhibited by SMAD3 silencing. Using a rat model of photoaging, our results show that the laser increased collagen expression and dermal thickness, demonstrating that the CO_2_ lattice laser has a profound therapeutic effect on photoaged skin. Together, our *in vitro* and *in vivo* data show that the CO_2_ lattice laser can reverse the skin aging caused by UVB, and indicate that this effect is mediated through SMAD3.

## INTRODUCTION

Skin photoaging is a complex process, which has many pathological similarities with skin injury. Histopathologically, it is mainly manifested by the decrease of collagen and the change of elastic fiber degeneration and deposition. External aging is regulated by complex environmental factors, including smoking, chemical exposure, and ultraviolet exposure [1, 2]. Ultraviolet irradiation reduces type I collagen through two mutually regulated pathways that stimulate collagen degradation and inhibit procollagen synthesis [3]. The decrease of type I procollagen synthesis induced by ultraviolet radiation is an important factor in the pathophysiology of skin photoaging [4]. Collagen remodeling is the most important process of skin regeneration in light injury; fibroblasts are the most important collagen-producing cells in collagen remodeling [1]. The UV radiation-induced photo-oxidation is the main damaging factor for the connective tissue of the skin. Yet, there is evidence that intrinsic and extrinsic aging processes have, at least in part, common biological, biochemical, and molecular mechanisms [5].

Cell therapy and laser therapy have been widely used in the treatment of photoaging skin. Fractional lasers, both ablative and non-ablative, are effective and safe, and have been used in a complementary manner for treating photoaged skin [6]. Conventional carbon dioxide (CO_2_) lattice laser is recognized as a “golden standard” measure to treat facial skin photoaging. It improves skin photoaging through the principle of focal photothermal action [7]. Fractional CO2 laser resurfacing has been used in the treatment of photoaging Asian population with a continued efficacy for up to 5 years [8].

Adult skin is composed of type I and III collagen; the type I collagen accounts for about 80% of skin collagen [9]. In dermis, type I collagen gathers into thick fibrous bundles parallel to the skin surface, interweaves into nets, and has a high mechanical stability. Type I collagen is an important component for maintaining skin tension, plumpness, and fullness [9]. Sunlight can affect type I collagen production, resulting in the reduction of mature collagen bundles, skin relaxation, and wrinkles. Other components in the matrix, such as aminopolysaccharides and proteoglycans, are also associated with photoaging [10].

The signaling protein SMAD3 promotes fibroblast proliferation, and induces synthesis and secretion of extracellular matrix proteins including collagen I. SMAD3 is down-regulated in photoaging cells, and is involved in skin photoaging [11]. In this study, we used primary human fibroblasts and a rat animal model to investigate the effect of CO2 lattice laser on skin photoaging.

## RESULTS

### CO_2_ lattice laser promotes fibroblast proliferation

**Figure 1:**
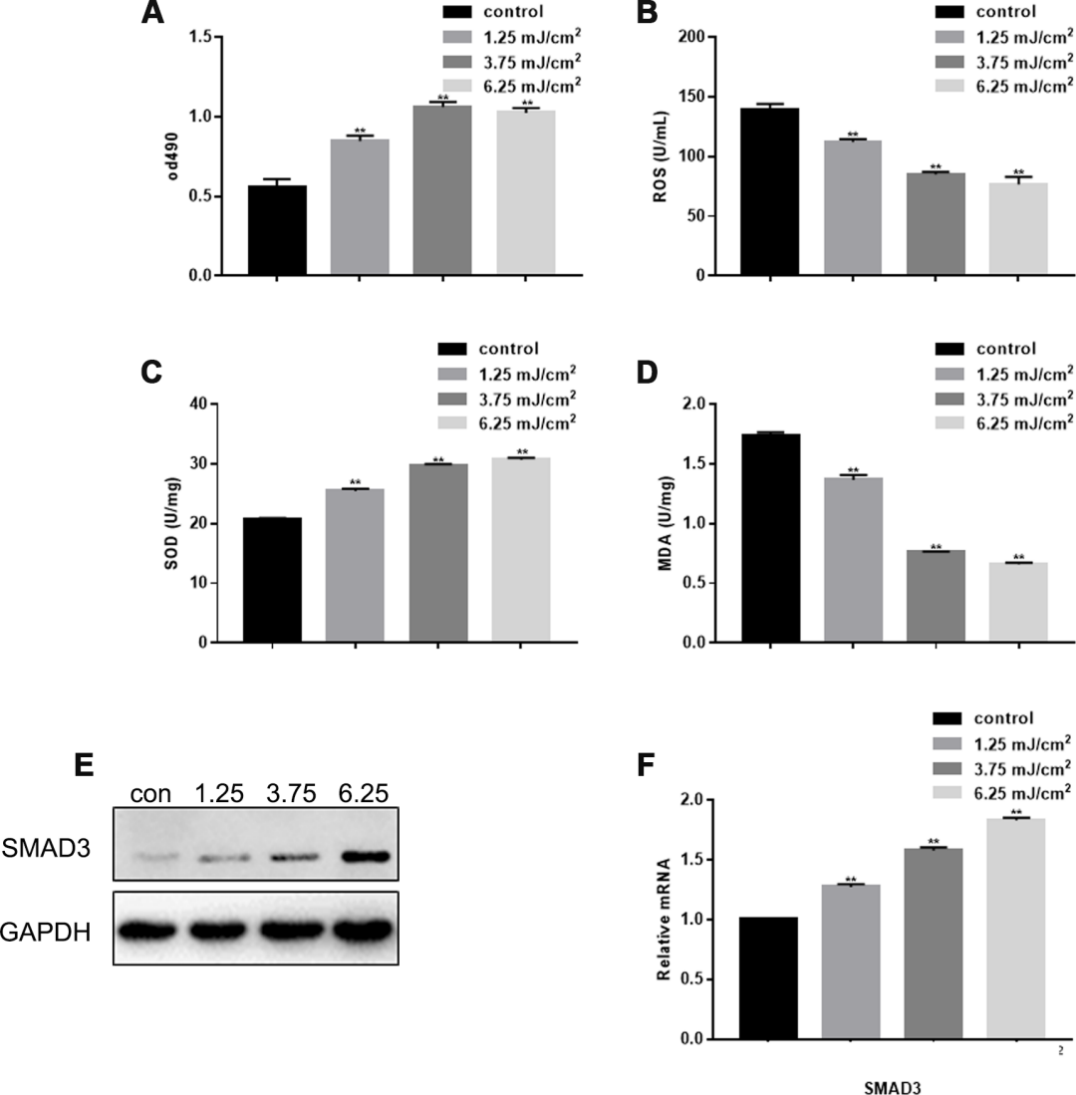
**Effect of CO_2_ Lattice Laser on Fibroblasts.** (**A**) Cell proliferation analyzed by MTT assay after treatment with 0, 1.25, 3.75 and 6.25 mJ/cm^2^ CO_2_ Lattice Laser. Data are shown as mean ± SEM.** P< 0.05 vs. 0 mJ/cm^2^ (**B**) ROS analyzed by ELISA in cells irradiated by CO_2_ lattice. Data are shown as mean ± SEM.** P< 0.05 vs. 0 mJ/cm^2^ (**C**) SOD expression analyzed by ELISA in cells irradiated by CO_2_ lattice laser. Data are shown as mean ± SEM.** P< 0.05 vs. 0 mJ/cm^2^ (**D**) MDA expression analyzed by ELISA in cells irradiated by CO2 lattice laser. Data are shown as mean ± SEM.** P< 0.05 vs. 0 mJ/cm^2^ (**E**, **F**) Western blotting and RT-PCR of SMAD3 in cells treated with 0, 1.25, 3.75 and 6.25 mJ/cm^2^ CO_2_ Lattice Laser; ** P< 0.05 vs. 0 mJ/cm^2^.

Using different doses of laser irradiation, MTT results showed that CO_2_ lattice laser could promote fibroblast proliferation, and the proliferation was enhanced with increasing laser dosage (Figure 1A). However, there was no significant difference in the proliferation ability between 3.75 mJ/cm^2^ irradiation group and 6.25 mJ/cm^2^ irradiation group. CO_2_ lattice laser also inhibited cell senescence by down-regulating production of reactive oxygen species (ROS) and malondialdehyde (MDA), and up-regulating expression of superoxide dismutase (SOD) (Figure 1B–1D). In addition, CO_2_ lattice laser increased gene and protein expression of SMAD3 (Figure 1E, 1F).

### CO_2_ lattice laser suppresses cell senescence induced by UVB

**Figure 2:**
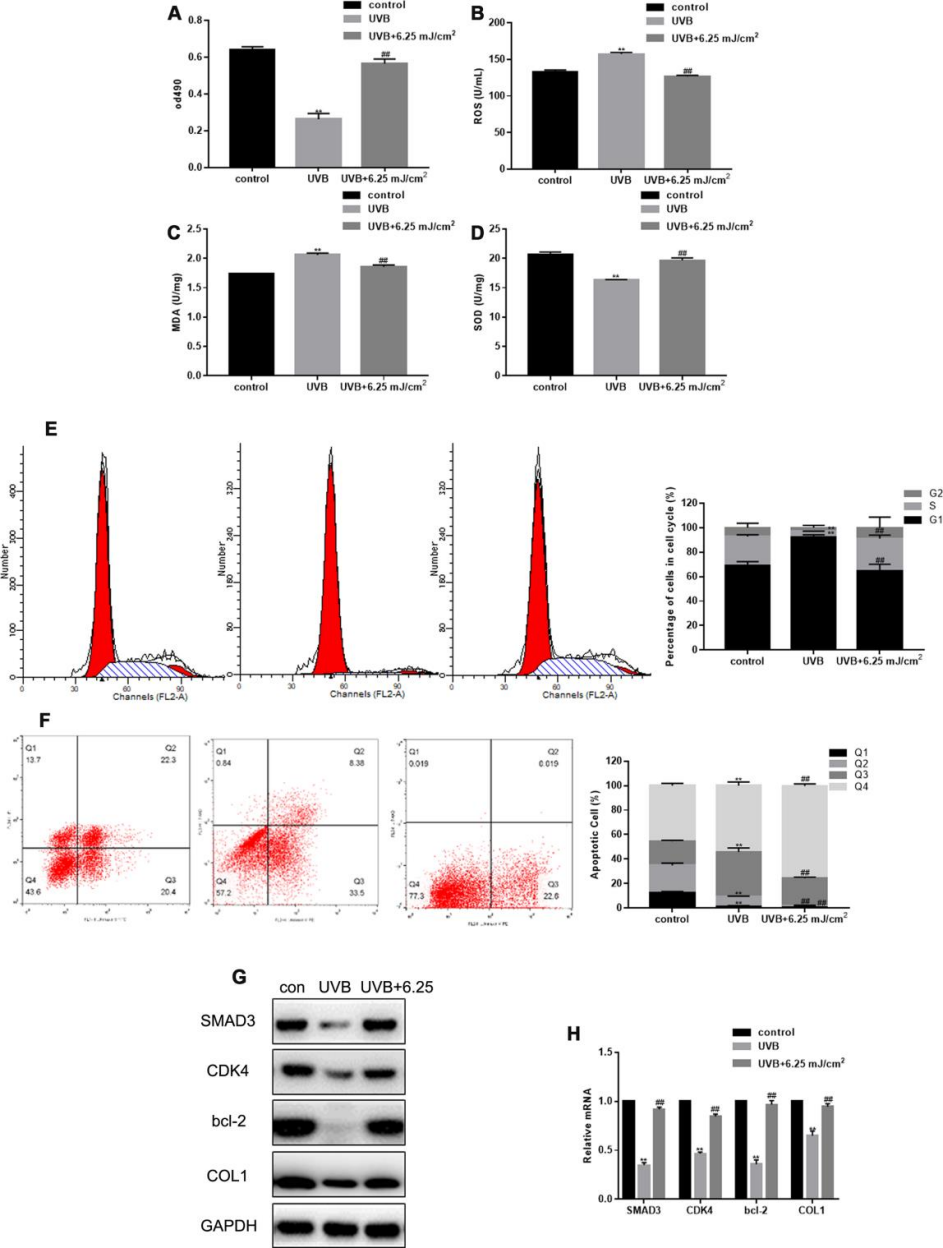
**CO_2_ lattice laser suppresses cell senescence induced by UVB.** (**A**) Proliferation of fibroblasts treated with control, UVB, or UVB and 6.25 mJ/cm^2^ CO_2_ Lattice Laser. Data are shown as mean ± SEM;** P< 0.05 vs. control, *## P*< 0.05 vs. UVB. (**B**) ELISA of ROS production in cells irradiated by control, UVB, or UVB and 6.25 mJ/cm^2^ CO_2_ Lattice Laser. Data are shown as mean ± SEM;** P< 0.05 vs. control, *## P*< 0.05 vs. UVB. (**C**) ELISA of SOD in cells irradiated by control, UVB or UVB and 6.25 mJ/cm^2^ CO_2_ Lattice Laser. Data are shown as mean ± SEM;** P< 0.05 vs. control, *## P*< 0.05 vs. UVB. (**D**) ELISA of MDA in cells irradiated by control, UVB or UVB and 6.25 mJ/cm^2^ CO_2_ Lattice Laser. Data are shown as mean ± SEM;** P< 0.05 vs. control, *## P*< 0.05 vs. UVB (**E**) Cell cycle analyzed by flow cytometry after treatment with control, UVB or UVB and 6.25 mJ/cm^2^ CO_2_ Lattice Laser. Data are shown as mean ± SEM.** P< 0.05 vs. control, *## P*< 0.05 vs. UVB. (**F**) Apoptosis was detected by Annexin V-PI after treatment with control, UVB or UVB and 6.25 mJ/cm^2^ CO_2_ Lattice Laser. Data are shown as mean ± SEM.** P< 0.05 vs. control, *## P*< 0.05 vs. UVB. (**G**, **H**) Expression of SMAD3, CDK4, Bcl-2, and COL1 analyzed by western blotting and RT-PCR. Data are shown as mean ± SEM;** P< 0.05 vs. control, *## P*< 0.05 vs. UVB.

As shown previously, UVB irradiation inhibited fibroblast proliferation, promoted expression of ROS and MDA, and inhibited expression of SOD (Figure 2A–2D). The inhibition of fibroblast proliferation and SOD expression, and the up-regulation of ROS and MDA induced by UVB irradiation were restored by CO_2_ lattice laser irradiation (Figure 2A–2D). In addition, UVB irradiation induced cell cycle arrest in G1 phase, and increased fibroblast apoptosis. However, the cell cycle arrest and apoptosis were reversed after CO_2_ lattice laser treatment (Figure 2E, 2F). Furthermore, CO_2_ lattice laser up-regulated gene and protein expression of SMAD3, CDK4, Bcl-2, and collagen 1 (COL1), which were down-regulated by UVB irradiation (Figure 2G, 2H).

### CO_2_ lattice laser reduces cell senescence through SMAD3

**Figure 3:**
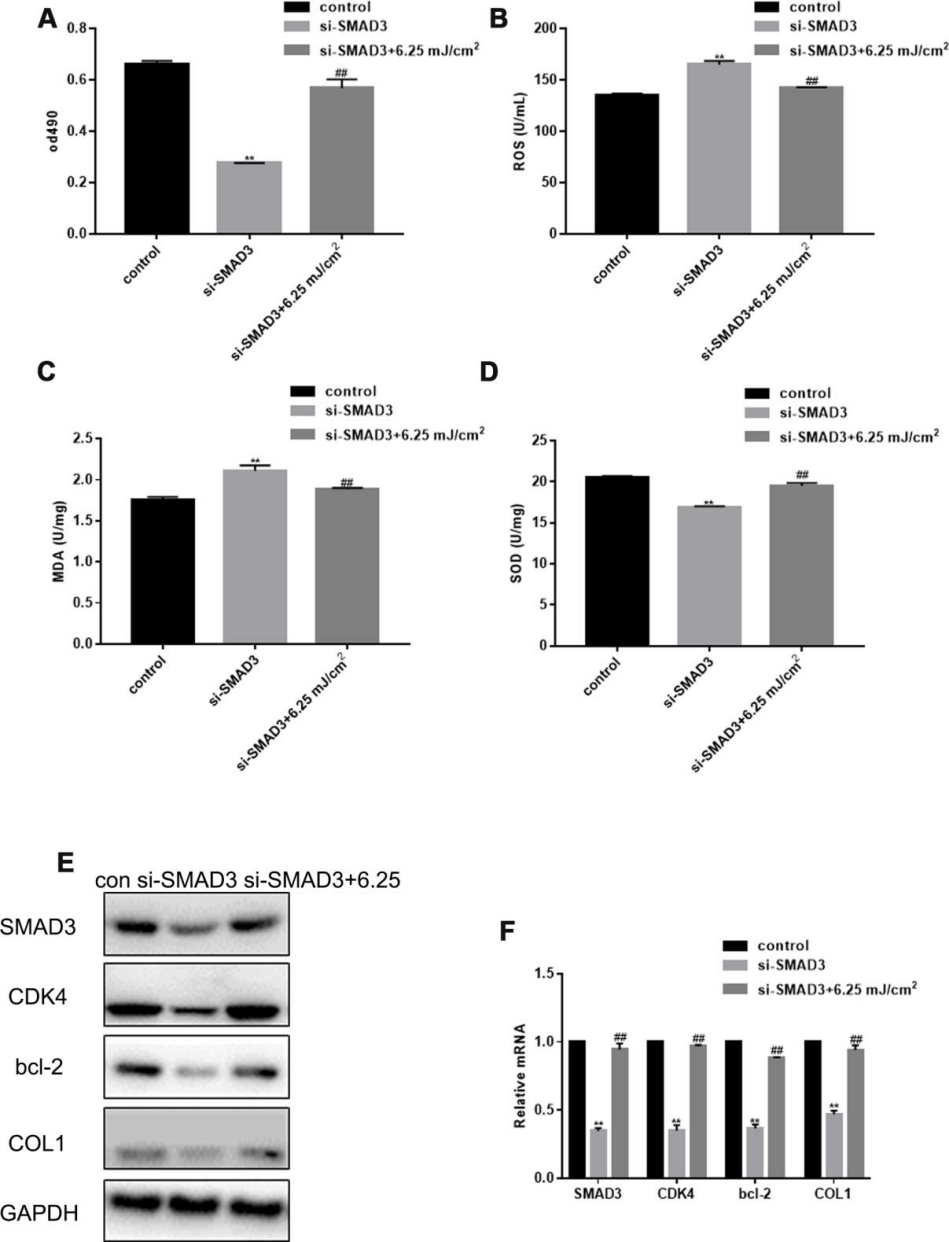
**CO2 Lattice Laser Reduces Cell Senescence through SMAD3.** (**A**) Proliferation of fibroblasts treated with control, si-SMAD3 or si-SMAD3 and 6.25 mJ/cm^2^ CO_2_ Lattice Laser. Data are shown as mean ± SEM.** P< 0.05 vs. control, *## P*< 0.05 vs. si-SMAD3. (**B**) ROS production in cells irradiated by control, si-SMAD3 or si-SMAD3 and 6.25 mJ/cm^2^ CO_2_ Lattice Laser. Data are shown as mean ± SEM.** P< 0.05 vs. control, *## P*< 0.05 vs. si-SMAD3. (**C**) MDA expression in cells irradiated by control, si-SMAD3 or si-SMAD3 and 6.25 mJ/cm^2^ CO_2_ Lattice Laser. Data are shown as mean ± SEM.** P< 0.05 vs. control, *## P*< 0.05 vs. si-SMAD3. (**D**) SOD expression in cells irradiated by control, si-SMAD3 or si-SMAD3 and 6.25 mJ/cm^2^ CO_2_ Lattice Laser. Data are shown as mean ± SEM.** P< 0.05 vs. control, *## P*< 0.05 vs. si-SMAD3. (**E**, **F**) Expression of SMAD3, CDK4, Bcl-2, and COL1 analyzed by western blotting and RT-PCR in cells treated with control, si-SMAD3 or si-SMAD3 and 6.25 mJ/cm^2^ CO_2_ Lattice Laser. Data are shown as mean ± SEM;** P< 0.05 vs. control, *## P*< 0.05 vs. si-SMAD3.

SMAD3 silencing inhibited fibroblast proliferation, but the proliferation was restored after CO_2_ laser irradiation (Figure 3A). SMAD3 silencing also increased the levels of ROS, up-regulated the expression of MDA, and down-regulated the expression of SOD; however, this effect was alleviated by CO_2_ lattice laser irradiation (Figure 3B–3D). In addition, CO_2_ lattice laser increased the expression of CDK4, Bcl-2, and COL1 induced by silencing SMAD3 (Figure 3E, 3F).

### Therapeutic effect of CO_2_ lattice laser on photoaging of rat skin

**Figure 4:**
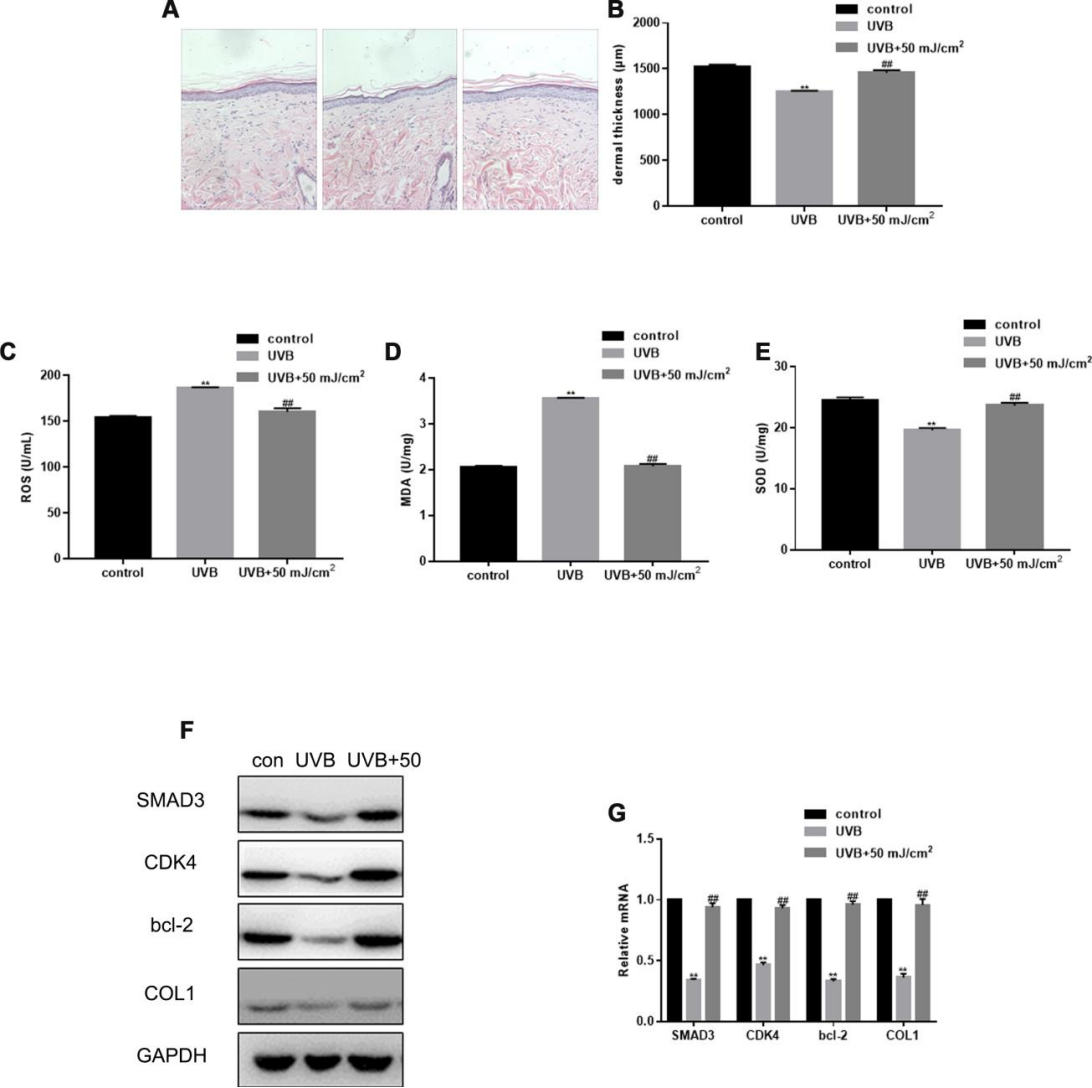
**Therapeutic effect of CO_2_ lattice laser on photoaging of rat skin.** (**A**) HE staining of skin structures in control, UVB irradiation, and CO_2_ lattice laser treatment groups. (**B**) Dermal thickness in control, UVB irradiation, and CO_2_ lattice laser treatment groups. Data are shown as mean ± SEM;** P< 0.05 vs. control, *## P*< 0.05 vs. UVB. (**C**) ROS production analyzed by ELISA in cells irradiated by control, UVB, or UVB and 50 mJ/cm^2^ CO_2_ Lattice Laser. Data are shown as mean ± SEM.** P< 0.05 vs. control, *## P*< 0.05 vs. UVB. (**D**) ELISA of MDA in cells irradiated by control, UVB or UVB and 50 mJ/cm^2^ CO_2_ Lattice Laser. Data are shown as mean ± SEM.** P< 0.05 vs. control, *## P*< 0.05 vs. UVB. (**E**) ELISA of SOD in cells irradiated by control, UVB or UVB and 50 mJ/cm^2^ CO_2_ Lattice Laser. Data are shown as mean ± SEM.** P< 0.05 vs. control, *## P*< 0.05 vs. UVB. **(F**, **G**) Expression of SMAD3, CDK4, Bcl-2, and COL1 analyzed by western blotting and RT-PCR in control, si-SMAD3 or si-SMAD3 and 6.25 mJ/cm^2^ CO_2_ Lattice Laser groups. Data are shown as mean ± SEM.** P< 0.05 vs. control, *## P*< 0.05 vs. UVB.

After UVB irradiation of rat skin, the dermis became thinner and the skin structure was disordered. In the control group, the structure of the epidermis was complete, the cell layers were clear, the thickness of the dermis was normal, the wavy fibrous tissues could be seen clearly, and the distribution was uniform and dense. However, after UVB irradiation, the epidermis was thickened with hyperkeratosis, had an unclear cell stratification, uneven distribution and disordered arrangement of cortical fibers, the diameter of collagen fibers increased, and inflammatory cells infiltrated the cross section. Importantly, in the treatment group, the thickness of skin and dermis significantly increased, and new, dense, and fine collagen fibers appeared in the superficial dermis, while some denatured and curly fibers were metabolized (Figure 4A, 4B). CO_2_ lattice laser treatment down-regulated the expression of ROS and MDA induced by UVB irradiation (Figure 4C, 4D). In addition, CO_2_ lattice laser treatment up-regulated the SOD expression reduced by UVB irradiation (Figure 4E). After CO_2_ lattice laser irradiation, the tissue expression of SMAD3, CDK4, Bcl-2, and COL1 significantly increased compared with the UVB irradiation group (Figure 4F, 4G).

## DISCUSSION

Photoaging refers to the aging process of skin caused by long-term exposure to ultraviolet light. Among the ultraviolet rays which cause photoaging, medium-wave ultraviolet light is especially damaging because of its high energy and strong penetration ability [14]. It can damage DNA, and induce expression of DNA damage repair factors and cell cycle regulatory proteins, resulting in a decreased fibroblast proliferation [15]. Exposure to UVA radiation can damage DNA and induce oxidative stress by generating ROS and MDA. SOD is a major antioxidant enzyme that provides a defense against the cell damage induced by oxidative stress [16]. UV can induce high concentration of ROS in the skin, resulting in photoaging [17]. In addition, MDA, a lipid peroxidation product, accumulates in the skin during aging, and mediates photoaging [18]. In contrast, SOD has an anti-aging effect [19]. In this study, we have evaluated the effect of UVB and CO_2_ laser on ROS, MDA, and SOD levels in skin cells during photoaging.

Previous studies have used cultured human primary fibroblasts to study the effect of UVB exposure on photoaging [20–22]. However, a single dose of UVB irradiation is difficult to control, and the effect greatly depends on the cell state. When the dosage is too high, cells undergo apoptosis; however, when the dosage is too low, cells can restore their normal functions through repair mechanisms [23]. Using a stable model of photoaging, our results show that UVB irradiation reduces fibroblast proliferation, blocks cell cycle in G1 phase, and increases apoptosis.

CO_2_ lattice laser is effective in repairing wrinkles and photoaging skin [24]. The combination of CO_2_ laser and RF(radio frequency) can be used to treat acne scars and various skin aging diseases [25–27]. It can promote epidermal reconstruction, dermal repair, and wound healing [28, 29]. Several recent studies have explored the therapeutic effect of laser on photoaging, but the mechanisms of its action are unknown [30]. CO_2_ and erbium: yttriumaluminum-garnet lasers, which can remove the stratum corneum, have been regarded as the gold standard for skin laser resurfacing. Fractional lasers combined with intense pulsed light therapy are expected to produce better efficacy with fewer side-effects for the treatment of photoaging skin [7]. A recent report has described the utility of a combined approach of fractional ablative CO_2_ with full-field erbium ablation for full face rejuvenation [31]. Rejuvenation with the fractional ablative carbon dioxide laser decreases the symptoms of facial aging in more than half of the patients [32]. Although many studies investigated the effect of CO_2_ laser on photoaging, most of them were clinical studies, and few of them analyzed the underlying mechanisms. In this study, we found that different doses of low concentration CO_2_ lattice laser irradiation could promote fibroblast proliferation.

Activation of SMAD3 promotes cell division and proliferation of fibroblasts, monocytes, and lymphocytes. SMAD3 induces collagen I synthesis, inhibits activation of matrix metalloproteinase, enhances expression of fibronectin, and reduces degradation of collagen fibers and extracellular matrix in fibroblasts [33]. In addition, it can stimulate fibroblast proliferation and inhibit fibroblast apoptosis by activating the MAPK pathway, thereby increasing the synthesis of collagen and elastic fibers [34]. This is the first study that focuses on the regulation of Smad3 by CO_2_ laser and UVB irradiation, and demonstrates that CO_2_ lattice laser irradiation promotes proliferation of human primary fibroblasts, down-regulates the levels of ROS and MDA, and induces the expression of SOD and SMAD3. In addition, our results show that CO_2_ lattice laser can up-regulate the expression of SMAD3, CDK4, Bcl-2, and COL1 down-regulated by UVB irradiation. CO_2_ lattice laser can reverse the aging of fibroblasts caused by UVB. Furthermore, CO_2_ lattice laser can reverse the cell senescence induced by SMAD3 silencing, indicating that the therapeutic effect of CO_2_ lattice laser is mediated by SMAD3.

The anti-aging effect of laser treatment can be achieved through epidermal reconstruction, dermal extracellular matrix regeneration, and remodeling [35]. This study shows that CO_2_ lattice laser can activate dermal fibroblasts, reduce UVB-induced cell cycle arrest and apoptosis, and stimulate collagen synthesis. Our HE staining results show that after the CO_2_ lattice laser treatment, the collagen expression increased, epidermis became thinner, dermis thickness increased, and inflammatory cell infiltration improved, demonstrating that the CO_2_ lattice laser treatment can have profound therapeutic effects on photoaging skin. In summary, our *in vitro* and *in vivo* data show that the CO_2_ lattice laser can reverse the skin aging caused by UVB, and indicate that this effect is mediated through SMAD3.

## MATERIALS AND METHODS

### Cells

Healthy skin tissues were obtained from 6 patients who underwent skin flap surgery in our hospital from May 2013 to May 2014. All patients signed the informed consent, and all procedures were approved by the Ethics Committee of the General Hospital of Northern Theater Command. The obtained healthy skin tissues were cut into fragments under aseptic conditions, and the epidermis and dermis were separated to obtain skin primary fibroblasts. Tissues were digested with type I collagenase and cultured in Dulbecco modified Eagle medium (DMEM, Hyclone, China) containing 10% fetal bovine serum (FBS, Hyclone, China), at 37 °C under 5 % CO_2_. The study was conducted using 2-5 generations of fibroblasts [12].

### Ultraviolet B (UVB) irradiation cell model

Primary fibroblasts with good cell viability and rapid proliferation were selected for the experiment. When the confluence was about 50%, the culture dish or six-well plate were taken out, the culture medium was discarded, cells were covered with PBS, and irradiated under UVB lamp tube at doses of 60 mJ/cm2. After each irradiation, DMEM medium was added. The interval time of irradiation was 12 h, 4 times in total.

### CO_2_ lattice laser therapy

Cells were treated with CO_2_ lattice laser (wavelength 10600 nm, spot size 9 mm * 9 mm, spot 0.125 mm, energy 0, 1.25, 3.75 and 6.25 mJ/cm^2^, frequency 5 Hz, repetition delay time 0.5 s). The experiment was carried out 24 h after irradiation.

### MTT assay

Cell proliferation was analyzed by the MTT tetrazolium assay. Cells were incubated with MTT solution for 4 h, and absorbance was measured at 490 nm.

### Reactive oxygen species (ROS), superoxide dismutase (SOD), and malondialdehyde (MDA) assays

ROS were determined by ROS kit, SOD was determined by SOD kit, and MDA was determined by MDA kit, according to the manufacturer’s instructions (Shanghai Renjie Biotechnology Co., Ltd., China, Shanghai).

### RNA isolation and real time PCR (RT-PCR)

RNA was extracted with Trizol, and converted to DNA using a reverse transcription kit (Roche, China, Beijing). SYBR kit (Roche Company, China, Beijing) was used for RT-PCR; a total of 35 cycles were carried out [13]. Primer sequences were as follows.

**Table:** 

**Name**	**Forward primer (5'->3')**	**Reverse primer (5'->3')**
SMAD3	CGGGACCTCACCGACTACCT	GGGCCGTGATCTCCTTCTG
CDK4	CATGTAGACCAGGACCTAAGG	GGAGGTCGGTACCAGAGTG
bcl-2	TCGCCCTGTGGATGACTG	GCTTGGCAATTAGTGGTC
COL1	GATGCCAATGTGGTTCGTG	TTCTTGCGGCTGCCCTCT
GAPDH	TGCGTGACATTAAGGAGAAG	CTGCATCCTGTCGGCAATG

### Western blotting

Proteins were extracted, separated by 10% polyacrylamide gel electrophoresis, and transferred to nitrocellulose membranes. After transfer, the membranes were blocked in 5% bovine serum albumin for 2 h at room temperature, and incubated overnight at 4°C with primary antibodies (SMAD3, CDK4, bcl-2, COL1 and GAPDH) (Santa Cruz, USA), followed by 1 h incubation with a secondary antibody (Santa Cruz, USA). Proteins were quantified by densitometry, and normalized to GAPDH.

### Cell cycle and apoptosis assays

For cell cycle analysis, cells were washed with PBS, fixed in ethanol, suspended in PI/RNase staining solution (Shanghai Biyuntian Biotechnology Co., Ltd., Shanghai, China), and analyzed by flow cytometry (FC).

For apoptosis assay, cells were washed with PBS, fixed in ethanol, suspended in Annexin V-PI staining solution (Shanghai Biyuntian Biotechnology Co., Ltd., Shanghai, China), and analyzed by FC.

### Animal model

Eighteen SD rats were randomly divided into a control group (6 rats), experimental group (6 rats), and treatment group (6 rats). The experimental and treatment groups were irradiated with UVB ultraviolet lamp (2 h/day, 60 days continuously). After 60 days, the animals in the treatment group were treated with a low energy CO_2_ lattice laser (wavelength 10600 nm, spot size 9 mm X 9 mm, spot 0.125 mm, energy 50 mJ/cm^2^, frequency 5 Hz, repetition delay time 0.5 s, coverage rate 10%) for two weeks. All groups were analyzed at the time of treatment completion in the treatment group.

### Hematoxylin-eosin (HE) staining

Tissues were fixed in formalin, embedded in paraffin, sliced, and stained with hematoxylin. After washing, the tissues were stained with eosin, and observed under a microscope (200-fold magnification).

### Statistical analysis

The data were analyzed by SPSS19.0 statistical analysis software. T test was used to evaluate the differences between the groups; P < 0.05 was considered significant.
